# Estimation of minimal clinically important change of the Japanese version of EQ-5D in patients with chronic noncancer pain: a retrospective research using real-world data

**DOI:** 10.1186/s12955-016-0438-2

**Published:** 2016-03-01

**Authors:** Kazutake Yoshizawa, Hisanori Kobayashi, Motoko Fujie, Yoshimasa Ogawa, Tsutomu Yajima, Koji Kawai

**Affiliations:** Medical Affairs Division, Janssen Pharmaceutical K.K., 3-5-2, Nishikanda, Chiyoda-ku, Tokyo, Japan; Japan Safety & Surveillance Division, Janssen Pharmaceutical K.K., 3-5-2, Nishikanda, Chiyoda-ku, Tokyo, Japan; Biostatistics Department, Quantitative Science Division, Janssen Pharmaceutical K.K., 3-5-2, Nishikanda, Chiyoda-ku, Tokyo, Japan; Sendai Pain Clinic Center, Medical Corporation Kanteikai, 99-1, Akai, Higashi Matsushima City, Sendai Japan

**Keywords:** Chronic pain, Minimal clinically important change, Numeric rating scale score, Japanese version EuroQol-5D

## Abstract

**Background:**

Quality of life (QoL) is routinely assessed and evaluated in medical research. However, in Japan, there is a lack of solid cutoff criteria for evaluating QoL improvement in chronic noncancer pain management. The present study was conducted to identify the minimal clinically important change (MCIC) of the Japanese version of EuroQol-5D 3L(EQ-5D) utility score and numeric rating scale (NRS) with an emphasis on chronic noncancer pain.

**Methods:**

The data source for this post hoc research was the post-marketing surveillance (PMS) data for a tramadol/acetaminophen combination tablet, which was previously conducted in real-world settings. The parameters extracted from the PMS data were sociodemographic characteristics, NRS, EQ-5D, and dichotomous physician’s global impression of treatment effectiveness (PGI). The optimal cutoff points of MCIC for EQ-5D utility and NRS scores were evaluated using receiver operating characteristics (ROC) analysis. An anchor-based approach using PGI was applied.

**Results:**

Data of 710 patients with chronic noncancer pain were extracted from the PMS database. The NRS score decreased by 2.7 (standard deviation, 2.3) points, whereas the EQ-5D score increased by 0*.*16 (0.20) points at 4 weeks from baseline. The changes from baseline in NRS and EQ-5D were significantly correlated (*r* = 0.53, *p* < 0.001). The estimated optimal cutoff points of MCIC for EQ-5D and NRS were 0.10 and −2.0 points, respectively. The area under the curve of ROC was > 0.80 in both analyses.

**Conclusion:**

These results demonstrated novel cutoff criteria for the Japanese version of EQ-5D, focusing on patients with chronic noncancer pain. The obtained criteria were fairly consistent and can be confidently utilized as an evaluation tool in medical research on chronic noncancer pain in Japan, with additional functionality and usability for QoL assessment in pain management practice.

**Trial registration:**

The data source of this post hoc research was a PMS study with the identifier number UMIN000015901 at umin.ac.jp, UMIN clinical trial registry (UMIN-CTR).

## Background

Chronic noncancer pain is one of the most common harmful experiences worldwide. It is not only responsible for impaired quality of life (QoL) and active daily life but is also associated with high costs because of long medical treatment and loss of work productivity [1, 2]. Among the Japanese population, 22.9 % suffer from chronic pain reported by numeric rating scale (NRS) to be a severity *≥* 5 and longer than 3 months duration. Of these patients, 55.9 % reported hospital visits due to pain, and 45.2 % of patients who had a hospital visit reported dissatisfaction with the care they received [3].

Health-related quality of life (HRQOL) instruments, particularly the EuroQol-5D (EQ-5D) [4], are widely used in health surveys and medical research. Lamers et al. showed the association between lower level EQ-5D utility scores and efficiency loss/absenteeism in individuals with low back pain [5]. Kovacs et al. pointed out a notable association between pain, disability, and QoL in patients with low back pain; however, they also postulated that clinically relevant improvements in pain severity exerted much less influence than did improvements in disability and QoL. This suggested that disability and/or QoL should be assessed when evaluating the effect of any form of treatment for low back pain [6]. In addition, these results suggested the importance of identifying target improvements in patients’ HRQOL and pain intensity and their association with success of chronic noncancer pain treatment. Several reports have suggested a minimal clinically important change (MCIC) of EQ-5D utility score in the treatment of low back pain [7–9]; moreover, several studies on musculoskeletal pain have reported MCIC of a pain intensity score [10–13]. However, no study has reported the MCIC of the Japanese version EQ-5D with an emphasis on chronic noncancer pain.

Although QoL assessment has become common in medical research in Japan, its interpretation still remains unclear. Suka et al. reported that the mean difference of EQ-5D utility scores between patients with low back pain and healthy volunteers was 0.09 in Japanese population [14], but MCIC was not evaluated in this study. A solid, methodologically sound, Japanese-specific MCIC of the EQ-5D utility score is essential for QoL assessment using the EQ-5D questionnaire in real-world management of chronic noncancer pain.

In the present study, we conducted a retrospective post hoc analysis to identify the optimal cutoff point (OCP) MCIC of the Japanese version EQ-5D utility score as well as the NRS score and to evaluate the association between the two scores using data from a multicenter post-marketing surveillance (PMS) of a tramadol/acetaminophen combination tablet in patients with chronic noncancer pain.

## Methods

### Data source

In this study, a post hoc data analysis was conducted using PMS data (data cutoff: September 2014, Identifier: UMIN000015901) collected from 1,316 patients with moderate chronic noncancer pain who were treated with a tramadol/acetaminophen combination tablet (tramadol 37.5 mg and acetaminophen 325 mg per tablet) in routine clinical practice [15]. PMS is a drug-specific registry that evaluates risks and benefits of a specific product. The PMS protocol was reviewed by internal review board members, including the ethical point of view, and was approved by the Japan Pharmaceuticals and Medical Devices Agency, Tokyo (https://www.pmda.go.jp/english/index.html). The PMS was conducted under the Japanese regulation (Ministry of Health, Labour and Welfare Ministerial Ordinance No. 171) of good post-marketing study practice (GPSP). All patients registered under the PMS received tramadol/acetaminophen combination tablets for up to 52 weeks based on regulatory approved label information in Japan. In Japan, tramadol/acetaminophen combination is indicated for patients with chronic noncancer pain that is unrelieved by non-opioid analgesic medication. The daily dose was determined at the discretion of each responsible practicing investigator. No restrictions were placed on concomitant medications or physical and psychological therapies.

### Measures and dataset

The sociodemographic data and name of the causal disease of pain were retrieved from PMS data. We also retrieved the NRS (0 to 10 points, 0: have no pain and 10: worst imaginable pain) for severity of the pain experienced by patients in the previous 24 h, the Japanese version of EQ-5D 3L [16] as patient-reported outcome measures, and the dichotomous physician’s global impression of treatment effectiveness (PGI; discrete choice of “effective” or “not effective”). The EQ-5D scale contains five dimensions assessing “mobility,” “self-care,” “usual activities,” “pain/discomfort,” and “anxiety/depression.” Each dimension has three levels: “no problem,” “some problem,” and “extreme problem.” The collected EQ-5D scale was translated into a global utility score by using a Japanese version of time trade-off value set. Higher EQ-5D global scores represent better QoL. While the original study collected data up to 52 weeks, relatively a small number of participant responded at 52 weeks. Since we intended to estimate the MCIC without any imputation of missing data, this might cause biased results. Therefore, because of the applicability of our results to the clinical practice and the sample size, the MCICs were estimated based on using data at week 4. Patients with complete EQ-5D, NRS, and PGI at baseline and Week 4 were included in the analyses.

### Statistical methods

Patient demographics were evaluated using descriptive statistics; categorical data were summarized by frequency and proportion, continuous data were summarized as mean with standard deviation (SD), median, maximum, and minimum.

A linear regression model was applied to evaluate correlation (r) and slope (β) between absolute changes from baseline of EQ-5D (dEQ-5D) and NRS (dNRS). The MCIC of EQ-5D and NRS at Week 4 were evaluated using receiver operating characteristic (ROC) curve anchored by PGI at Week 4. An area under the ROC curve (AUC) > 0.70 was considered as a sufficient level of prediction to identify MCIC [17]. MCIC, the OCP of ROC, was determined using the maximal point of the sum of sensitivity and specificity [18]. Subsequently, the ratio of patients who achieved MCIC of EQ-5D and NRS was calculated. In addition, logistic regression analysis was performed to investigate the influence of dNRS and dEQ-5D on PGI judgment. Standardized (Z score) dNRS and dEQ-5D were utilized for this logistic regression analysis in terms of adjustment to make the mean equal to zero and the SD equal to 1.0. The formulae for calculating standardized dNRS and dEQ-5D in each patient are

- ▪ (dNRS in each patient − mean of dNRS)/SD of dNRS
- ▪ (dEQ-5D in each patient − mean of dEQ-5D)/SD of dEQ-5D

A two-sided *p* value < 0.05 was considered statistically significant. All statistical analyses were performed using R version 3.2.1 (Foundation for Statistical Computing, Vienna, Austria) and JMP 9.0 (SAS Institute Japan Ltd. Tokyo, Japan).

## Results

### Descriptive analysis of demographics

Data from a total of 710 patients with complete EQ-5D, NRS, and PGI at baseline and Week 4 were retrieved and classified into the post hoc analysis set. Table 1 presents the demographics of these patients. The mean age was 66.7 (SD, 14.4) years and a majority of patients were women (61.7 %). The mean duration of pain symptoms was 2.0 (3.4) years, the mean NRS score at baseline was 7.1 (1.9), and the mean EQ-5D utility score was 0.5 (0.2). Patients were frequently diagnosed with a musculoskeletal/orthopedic disorder, and postherpetic neuralgia (PHN) was included as typical neuropathic pain (Table 1).

**Table 1:** Patient demographics

	*n*	mean [SD] or *n* (%)
Gender		
Male	710	272 (38*.*3)
Female	710	438 (61*.*7)
Age (years: mean [SD])	710	66*.*7 [14*.*4]
BMI (kg/m^2^: mean [SD])	372	23*.*4 [3*.*8]
Disease Duration (years: mean [SD])	499	2*.*0 [3*.*4]
Baseline NRS (score: mean [SD])	710	7*.*1 [1*.*9]
Baseline EQ-5D (score: mean [SD])	710	0*.*5 [0*.*2]
Major the causal disease of pain (multiple allowed)		
Osteoarthritis	710	182 (25*.*6)
Lumbago	710	285 (40*.*1)
Rheumatoid arthritis	710	29 (4*.*1)
Neck, shoulder, and arm syndrome	710	44 (6*.*2)
Postherpetic neuralgia	710	54 (7*.*6)
Periarthritis scapulohumeralis	710	46 (6*.*5)
Spinal stenosis	710	61 (8*.*6)
Disk herniation	710	30 (4*.*2)
Others	710	233 (32*.*8)
Site of pain		
Head	710	26 (3*.*7)
Lumbar region	710	374 (52*.*7)
Lower extremity	710	264 (37*.*2)
Cervical region	710	90 (12*.*7)
Shoulder	710	101 (14*.*2)
Upper extremity	710	54 (7*.*6)
Back	710	59 (8*.*3)
Chest/abdomen	710	34 (4*.*8)
Hand/foot	710	55 (7*.*7)

BMI denotes body mass index, *NRS* numeric rating scale, *EQ-5D* EuroQol 5D, *SD* standard deviation

### Mean change from baseline and correlations

Table 2 summarizes the mean change from baseline in NRS and EQ-5D at 4 weeks after treatment. The mean ± SD dNRS and dEQ-5D from baseline were −2.7 *±* 2*.*3 points (paired *t*-test, *p* < 0.001) and 0*.*16 *±* 0*.*20 points (paired *t*-test, *p* < 0.001), respectively. Larger mean dNRS was observed in lumbago (−2.9) and osteoarthritis (−2.9), whereas a relatively smaller change was observed in rheumatoid arthritis (−1.7). The largest improvement of EQ-5D was observed in patients with lumbago (0.19), and the smallest improvement was observed in those with rheumatoid arthritis (0.10). The proportion of patients with PGI assessed as “effective” was 86.1 % (95 % CI, 83.9–88.3) at Week 4.

**Table 2:** PGI and mean changes from baseline in NRS and EQ-5D utility scores

	n	PGI Effective	dNRS Week 4 (∆)		*p*	dEQ-5D Week 4 (∆)		*p*
			Baseline	Week 4 (∆)		Baseline	Week 4 (∆)	
Overall	710	614 (86*.*5)	7*.*1 [1*.*9]	*−*2*.*7 [2*.*3]	<0.0001	0*.*51 [0*.*20]	0*.*16 [0*.*20]	<0.0001
Major the causal disease of pain (multiple allowed)								
Osteoarthritis	182	168 (92*.*3)	7*.*0 [2*.*0]	*−*2*.*9 [2*.*3]	<0.0001	0*.*53 [0*.*15]	0*.*17 [0*.*19]	<0.0001
Lumbago	285	253 (88*.*8)	7*.*4 [1*.*8]	*−*2*.*9 [2*.*4]	<0.0001	0*.*47 [0*.*23]	0*.*19 [0*.*22]	<0.0001
Rheumatoid arthritis	29	21 (72*.*4)	6*.*4 [2*.*1]	*−*1*.*7 [2*.*0]	0.0002	0*.*51 [0*.*18]	0*.*10 [0*.*15]	0.0018
Neck, shoulder and arm syndrome	44	38 (86*.*4)	7*.*5 [1*.*7]	*−*2*.*8 [2*.*2]	<0.0001	0*.*61 [0*.*14]	0*.*12 [0*.*18]	<0.0001
Postherpetic neuralgia	54	46 (85*.*2)	6*.*7 [2*.*1]	*−*2*.*9 [2*.*1]	<0.0001	0*.*58 [0*.*17]	0*.*15 [0*.*16]	<0.0001
Periarthritis scapulohumeralis	46	39 (84*.*8)	7*.*4 [1*.*8]	*−*2*.*2 [1*.*7]	<0.0001	0*.*55 [0*.*13]	0*.*12 [0*.*16]	<0.0001
Spinal stenosis	61	47 (77*.*0)	7*.*1 [2*.*0]	*−*2*.*5 [2*.*6]	<0.0001	0*.*53 [0*.*10]	0*.*12 [0*.*16]	<0.0001
Disk herniation	30	27 (90*.*0)	6*.*8 [1*.*8]	*−*2*.*7 [2*.*4]	<0.0001	0*.*50 [0*.*19]	0*.*17 [0*.*20]	<0.0001
Others	233	196 (84*.*1)	6*.*9 [2*.*0]	*−*2*.*5 [2*.*6]	<0.0001	0*.*52 [0*.*18]	0*.*13 [0*.*20]	<0.0001

dEQ-5D denotes change in EuroQol-5D, *dNRS* change in numeric rating scale, *PGI* physician’s global impression

**Fig. 1:**
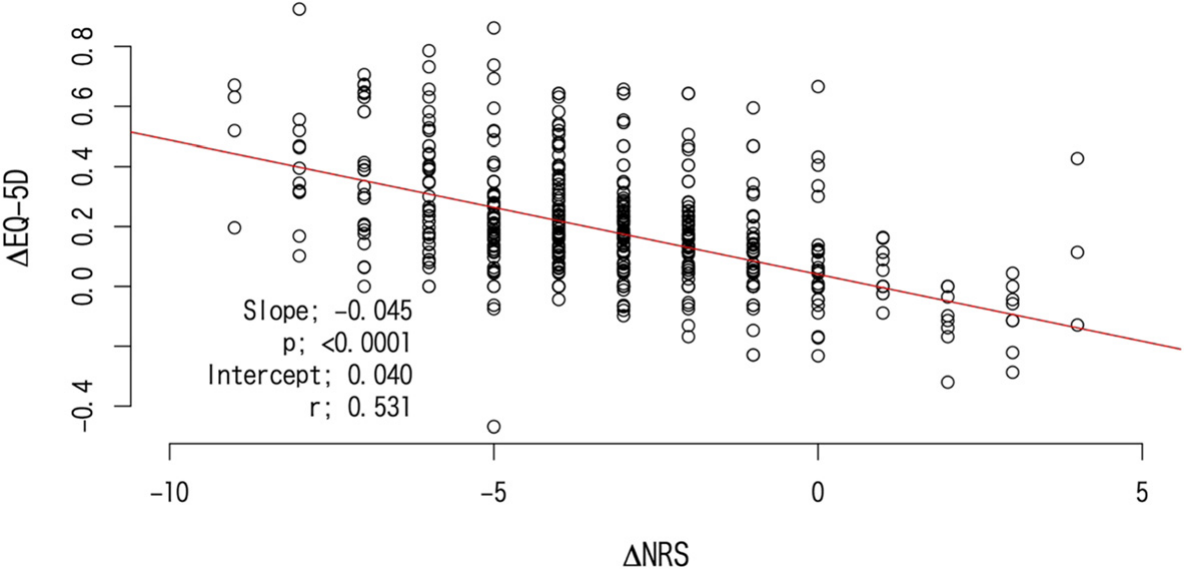
Correlation between dNRS (ΔNRS) and dEQ-5D utility score (ΔEQ-5D)

Table 3 presents the correlation between absolute dNRS and dEQ-5D. Significant correlations were observed between the two scores (Fig. 1, Table 3). There was a relatively close negative correlation between the dNRS and dEQ-5D at Week 4, with a correlation coefficient (r) of 0.53 (*p* < 0.0001). The simple linear regression model indicated that for each 1-point reduction in NRS, the EQ-5D score is expected to improve by 0.045 (*m*). When stratified by causal disease, none of the groups showed zero slope, but the lumbago, rheumatoid arthritis, and PHN subgroups showed smaller Pearson correlations (r < 0.5).

**Table 3:** Correlation Between dEQ-5D Utility Score (y) and dNRS Δ (x) by Causal Disease

	n	Slope (β)	Pearson’s correlation coefficient (r)	*p*
Overall	710	−0.045	0.531	<0.0001
Major the causal disease of pain (multiple allowed)				
Osteoarthritis	182	−0.044	0.542	<0.0001
Lumbago	285	−0.042	0.457	<0.0001
Rheumatoid arthritis	29	−0.037	0.498	0.0060
Neck, shoulder and arm syndrome	44	−0.046	0.552	0.0001
Postherpetic neuralgia	54	−0.037	0.468	0.0004
Periarthritis scapulohumeralis	46	−0.057	0.619	<0.0001
Spinal stenosis	61	−0.041	0.660	<0.0001
Disk herniation	30	−0.057	0.698	<0.0001
Others	233	−0.046	0.601	<0.0001

dEQ-5D denotes change in EuroQol-5D, *dNRS* change in numeric rating scale

### Estimated MCIC of dEQ-5D, dNRS, and achievement ratio of MCIC

**Fig. 2:**
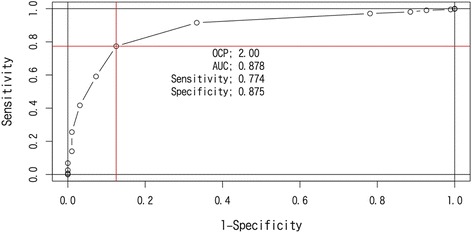
ROC curve of PGI and change from baseline NRS (dNRS)

**Fig. 3:**
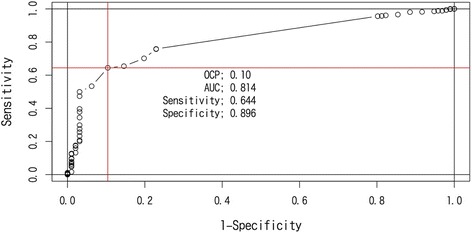
ROC curve of PGI and change from baseline EQ-5D (dEQ-5D)

The overall MCIC of ROC analysis based on PGI showed a cutoff point of −2.0 points (AUC = 0.88, sensitivity = 0.77, specificity = 0.88) for dNRS; for dEQ-5D, it was 0.10 (AUC = 0.88, sensitivity = 0.64, specificity = 0.90) (Figs. 2 and 3). The specificity of both of dNRS and dEQ-5D were similar but the sensitivity of dNRS was higher than dEQ-5D. The ratio of MCIC achievement and PGI “effective” classification are shown in Table 4. In total, 405 (57.0 %) patients had improved EQ-5D ≥ 0.10 points, whereas 487 (68.6 %) patients had improved NRS ≥ 2.0 (Table 4).

**Table 4:** Rate of MCIC Achievement

	PGI Effective	NRS *≤ −*2	EQ-5D *≥* 0*.*10
Overall	614 (86.5)	487 (68.6)	405 (57.0)
Major the causal disease of pain (multiple allowed)			
Osteoarthritis	168 (92.3)	130 (71.4)	113 (62.1)
Lumbago	253 (88.8)	214 (75.1)	177 (62.1)
Rheumatoid arthritis	21 (72.4)	18 (62.1)	13 (44.8)
Neck, shoulder and arm syndrome	38 (86.4)	32 (72.7)	22 (50.0)
Postherpetic neuralgia	46 (85.2)	39 (72.2)	30 (55.6)
Periarthritis scapulohumeralis	39 (84.8)	33 (71.7)	21 (45.7)
Spinal stenosis	47 (77.0)	37 (60.7)	26 (42.6)
Disk herniation	27 (90.0)	18 (60.0)	17 (56.7)
Others	196 (84.1)	142 (60.9)	112 (48.1)

EQ-5D denotes EuroQol-5D, *MCIC* minimal clinically important change, *NRS* numeric rating scale, *PGI* physician’s global impression

### Coefficient of NRS and EQ-5D to PGI judgment

Logistic regression analysis by using standardized dNRS and dEQ-5D as covariates was conducted to further investigate the impact of dNRS and dEQ-5D on PGI judgment. The absolute value of standardized coefficient is compatible between the dNRS and dEQ-5D, and the coefficient of NRS was higher (−1.490, *p* < 0.001) than the coefficient of EQ-5D (0.795, *p* < 0.001).

## Discussion

In our study population, the most common cause of pain was musculoskeletal disorders. In a majority of patients, baseline NRS scores corresponding to pain intensity could be qualified as moderate-to-severe pain. While Nawata et al. had reported an average EQ-5D utility score of 0.83 (95 % CI, 0.82–0.84; *n* = 2937) in Japanese elderly (mean age, 72.3 [SD, 6.1] years) volunteers [19], the mean EQ-5D utility score at baseline in our study population (mean age, 66.7 [SD, 14.4] years) was remarkably lower (0.51 [SD, 0.20])

In general, pain intensity scores are associated with QOL scale in pain disorders [6, 20, 21]. In our results, a significant correlation (*r* = 0.53, *p* < 0.001) was observed between dNRS and dEQ-5D scores, and both dNRS and dEQ-5D supported the treatment effect of tramadol/acetaminophen therapy. The simple linear regression model indicated that a 1-point reduction in dNRS corresponded to a 0.045-point improvement in dEQ-5D. This conversion ratio may be applicable to clinical practice for chronic pain management when practitioners evaluate clinical outcomes using the NRS or EQ-5D scales. However, there is a possibility that other factors strongly associate with the change of NRS and EQ-5D. For example, an individual patient who had severe side effects would result in a deterioration of QoL. Therefore we need to evaluate QoL and functionality along with pain severity in the treatment of chronic pain patients.

The primary aim of this study was to estimate the OCP for MCIC of the NRS and the Japanese version of EQ-5D, with an emphasis on noncancer chronic pain using simple and conventional statistical analysis methods. The OCP of MCIC was estimated to be a 0.10-point increase in EQ-5D and a 2.0-point reduction in NRS.

Two-thirds of physicians have agreed on an OCP of 2- to 4-point reduction in NRS scores as the MCIC [22]. Salaffi et al. reported a 2-point reduction in NRS as the minimal treatment target for chronic pain [10]. The mean change in the pain intensity score from baseline by using a 10-mm visual analog scale at 4 weeks after tramadol/acetaminophen therapy was −22.35 mm (SD, 21.19) in the clinical registration trial in Japanese patients with chronic noncancer pain [23]. These results support that the MCIC of NRS we propose is clinically relevant in the Japanese population. In addition, several reports have suggested a minimal clinically important difference in EQ-5D utility score as approximately 0.08 [7] to 0.17 [8] in the treatment of low back pain. The results of our study that used Japanese real-world data are consistent with these previous reports. We identified an MCIC that is clinically comparable to that derived from medical research conducted in multi-countries and that may provide the reasonable minimum treatment target for Japanese patients with chronic pain in real-world practice. The distribution-based approach using one- half of standard deviation at baseline value was frequently used to determine the OCP of MCIC [24]. In this study, one- half of standard deviation at baseline of EQ-5D was 0.1 (Table 2) that was same as OCP of MCIC produced by the anchor- based approach using PGI. We did not see the discrepancy between the conclusions of both approaches. As a limitation of our result, the estimated MCICs were based on the analysis of complete data at baseline and week 4. We assumed missing at random for missing data mechanism and no imputation for missing data has been performed.

The PGI (effectiveness) and the rate of MCIC achievement of EQ-5D (<0.1) and NRS (< −2.0) were 86.5 % (614/710), 57.0 % (405/710), and 68.6 % (487/710), respectively. The rate of MCIC achievement in both patient-reported outcomes was lower and seemed more conservative than physician’s judgment. A larger discrepancy was observed between physician’s judgment and the ratio of MCIC achievement in EQ-5D than in NRS. In addition, logistic regression analysis using standardized dNRS and dEQ-5D suggested that improvements in both the NRS score (*p* < 0.001) and the EQ-5D utility score (*p* < 0.001) were independently associated with a more favorable PGI judgment. The absolute value of the standardized coefficient was compatible between dNRS and dEQ-5D, but the coefficient of dNRS was higher (−1.490) than the coefficient of dEQ-5D (0.795), suggesting that physician’s judgment is influenced more by changes in NRS scores than by changes in EQ-5D utility scores. This result was consistent with the result of ROC analysis that the sensitivity of OPC of MCIC on dNRS was higher than dEQ-5D. This may suggest that when judging improvement, physicians should consider improvements in pain severity than improvements in the patient’s QoL.

## Conclusion

The post treatment changes from baseline in NRS scores and Japanese version of EQ-5D utility scores were well correlated in Japanese patients with chronic noncancer pain. Simple ROC using the PGI-anchored approach estimated a 0.10-point increase as the OCP of MCIC in EQ-5D utility scores and a 2.0-point reduction in NRS scores. Our results demonstrated novel cutoff criteria for the Japanese version of EQ-5D, focusing on patients with chronic noncancer pain. The obtained criteria were fairly consistent and could be utilized as an evaluation tool in medical research on noncancer chronic pain in Japan, providing additional functionality and usability for QoL assessment in pain management practice. The EQ-5D scale is a useful evaluation tool for QoL assessment in real-world treatment of Japanese patients with chronic noncancer pain.

### Ethics approval and consent to participate

Not applicable

## Abbreviations

AUC: Area under the curve
EQ-5D: EuroQol-5D
GPSP: Good post-marketing surveillance practice
HRQOL: Health-related quality of life
MCIC: Minimal clinically important change
NRS: Numeric rating scale
OCP: Optimal cutoff point
PGI: Physician’s global impression
PHN: Postherpetic neuralgia
PMS: Post-marketing surveillance
QOL: Quality of life
ROC: Receiver operating characteristic
SD: Standard deviation

## References

[1] Murphy TM (2000). Chronic Pain.

[2] Gaskin DJ, Richard P (2012). The economic costs of pain in the United States. J Pain.

[3] Matsudaira K, Takeshita K, Kunogi J (2011). Pain associated cross-sectional epidemiological (PACE) survey 2009. JP Pain Clinic.

[4] Group EQ (1990). EuroQol - a new facility for the measurement of health-related quality of life. Health Policy.

[5] Lamers LM, Meerding WJ, Severens JL, Brouwer WB (2005). The relationship between productivity and health-related quality of life: an empirical exploration in persons with low back pain. Qual Life Res.

[6] Kovacs FM, Abraira V, Zamora J, Real MT, Llobera J, Fernandez C (2004). Correlation between pain, disability, and quality of life in patients with common low back pain. Spine.

[7] Walters SJ, Brazier JE (2005). Comparison of the minimally important difference for two health state utility measures: EQ-5D and SF-6D. Qual Life Res.

[8] Johnsen G, Hellum C, Nygaard O (2013). Comparison of the SF6D, the EQ5D, and the oswestry disability index in patients with chronic low back pain and degenerative disc disease. Musculoskeletal Disorders.

[9] Scott LP, Owoicho A, Alexander R (2011). Utility of minimum clinically important difference in assessing pain, disability, and health state after transforaminal lumbar interbody fusion for degenerative lumbar spondylolisthesis. J Neurosurg Spine.

[10] Salaffi F, Stancati A, Silvestri C (2004). Minimal clinically important changes in chronic musculoskeletal pain intensity measured on a numeric rating scale. Eur J Pain.

[11] van der Roer N, Ostelo RW, Bekkering GE, van Tulder MW, de Vet HC (2006). Minimal clinically important change for pain intensity, functional status, and general health status in patients with nonspecific low back pain. Spine.

[12] Ostelo RW, Deyo RA, Stratford P, Waddell G, Croft P, Von Korff M (2008). Interpreting change scores for pain and functional status in low back pain towards International consensus regarding minimal important change. Spine.

[13] Francisco MK, Víctor A, Ana R, Josep C, Luis A (2008). Minimum detectable and minimal clinically important changes for pain in patients with nonspecific neck pain. BMC Musculoskelet Disord.

[14] Suka M (2009). Cost-effectiveness of preventive interventions against back pain in Japanese health examinees. Health Evaluation and Promotion.

[15] Yoshizawa K, Kawai K, Fujie M, Suzuki J, Ogawa Y, Yajima T (2015). Overall safety profile and effectiveness of tramadol hydrochloride/acetaminophen in patients with chronic noncancer pain in Japanese real-world practice. Curr Med Res Opin.

[16] EuroQol Translation Team J (1998). The development of Japanese EuroQol instrument. Iryo to Shakai.

[17] Hanley JA, McNeil BJ (1982). The meaning and use of the area under a receiver operating characteristic (ROC) curve. Radiology.

[18] de Vet HC, Ostelo RW, Terwee CB, Van der Roer N, Knol DL, Beckerman H (2007). Minimally important change determined by a visual method integrating an anchor-based and a distribution-based approach. Qual Life Res.

[19] Nawata S, Yamada Y, Ikeda S (2000). EuroQol Study of the Elderly General Population: Relationship with IADL and Other Attributes. Healthcare and Society.

[20] Yazdi-Ravandi S, Taslimi Z, Jamshidian N, Saberi H, Shams J, Haghparast A (2013). Prediction of quality of life by self-efficacy, pain intensity and pain duration in patient with pain disorders. Basic Clin Neurosci.

[21] Kim W, Jin YS, Lee CS, Bin SI, Lee SY, Choi KH (2015). Influence of knee pain and low back pain on the quality of life in adults older than 50 years of age. PMR.

[22] Varrassi G, Müller-Schwefe G (2012). The international CHANGE PAIN physician survey: does specialist influence the perception of pain and its treatment?. Curr Med Res Opin.

[23] Inoue Y, Nishimura A, Taguma K, Sakata H (2012). A phase III, open-label, long-term clinical trial of tramadol hydrochloride/acetaminophen combination tablet in patients with chronic pain. Journal of Joint Surgery.

[24] Le QA, Doctor JN, Zoellner LA, Feeny NC (2013). Minimal clinically important differences for the EQ-5D and QWB-SA in post-traumatic stress disorder (PTSD): result from a doubly randomized preference trial (DRPT). Health Qual Life Outcomes.

